# Molecular characterization and evolution of a gene family encoding male-specific reproductive proteins in the African malaria vector *Anopheles gambiae*

**DOI:** 10.1186/1471-2148-11-292

**Published:** 2011-10-06

**Authors:** Emiliano Mancini, Francesco Baldini, Federica Tammaro, Maria Calzetta, Aurelio Serrao, Phillip George, Isabelle Morlais, Daniel Masiga, Igor V Sharakhov, David W Rogers, Flaminia Catteruccia, Alessandra della Torre

**Affiliations:** 1 Istituto-Pasteur - Fondazione Cenci Bolognetti, Dipartimento di Sanità Pubblica e Malattie Infettive, Sapienza Università di Roma, Rome, Italy; 2 Dipartimento di Medicina Sperimentale e Scienze Biochimiche, Università di Perugia, Terni, Italy; 3 Department of Entomology, Virginia Tech, Blacksburg, VA, USA; 4 Laboratoire d'entomologie médicale, OCEAC-IRD, BP288, Yaoundé, Cameroon; 5 Molecular Biology and Bioinformatics Unit, International Centre of Insect Physiology and Ecology, Nairobi, Kenya; 6 Max-Planck Institute for Evolutionary Biology, Plön, Germany; 7 Division of Cell and Molecular Biology, Imperial College London, London, UK

## Abstract

**Background:**

During copulation, the major Afro-tropical malaria vector *Anopheles gambiae *s.s. transfers male accessory gland (MAG) proteins to females as a solid mass (i.e. the "mating plug"). These proteins are postulated to function as important modulators of female post-mating responses. To understand the role of selective forces underlying the evolution of these proteins in the *A. gambiae *complex, we carried out an evolutionary analysis of gene sequence and expression divergence on a pair of paralog genes called *AgAcp34A-1 *and *AgAcp34A-2*. These encode MAG-specific proteins which, based on homology with *Drosophila*, have been hypothesized to play a role in sperm viability and function.

**Results:**

Genetic analysis of 6 species of the *A. gambiae *complex revealed the existence of a third paralog (68-78% of identity), that we named *AgAcp34A-3*. FISH assays showed that this gene maps in the same division (34A) of chromosome-3R as the other two paralogs. In particular, immuno-fluorescence assays targeting the C-terminals of *AgAcp34A-2 *and *AgAcp34A-3 *revealed that these two proteins are localized in the posterior part of the MAG and concentrated at the apical portion of the mating plug. When transferred to females, this part of the plug lies in proximity to the duct connecting the spermatheca to the uterus, suggesting a potential role for these proteins in regulating sperm motility. *AgAcp34A-3 *is more polymorphic than the other two paralogs, possibly because of relaxation of purifying selection. Since both unequal crossing-over and gene conversion likely homogenized the members of this gene family, the interpretation of the evolutionary patterns is not straightforward. Although several haplotypes of the three paralogs are shared by most *A. gambiae *s.l. species, some fixed species-specific replacements (mainly placed in the N- and C-terminal portions of the secreted peptides) were also observed, suggesting some lineage-specific adaptation.

**Conclusions:**

Progress in understanding the signaling cascade in the *A. gambiae *reproductive pathway will elucidate the interaction of this MAG-specific protein family with their female counterparts. This knowledge will allow a better evaluation of the relative importance of genes involved in the reproductive isolation and fertility of *A. gambiae *species and could help the interpretation of the observed evolutionary patterns.

## Background

Across many taxa, genes encoding proteins involved in reproductive processes often evolve rapidly and can contribute to the establishment of barriers to fertilization that might ultimately lead to speciation [1,2]. Male seminal fluid proteins transferred during mating induce a series of physiological and behavioral changes in females, generally referred to as post-mating responses [3]. In *Drosophila *species, male accessory gland proteins (Acps) transferred to females alongside sperm are known to modulate female physiology after mating and induce ovulation, oogenesis, sperm storage and a temporary refractoriness to further mating. Many *Drosophila *Acps evolve rapidly because of positive selection due to sexual conflict [4-12]. Among them, the "sex peptide" *Acp70A *has been shown to be one of the most divergent genes in the *Drosophila *genome [4,6] and to be partly responsible for species-specific usage of gametes [13]. Studies on the *D. melanogaster *and *D. pseudoobscura *groups have also shown that Acps are frequently subject to gene duplication [10,14], a mechanism that allows the acquisition of new genes and plays a substantial role in the diversification of closely related species [15].

*Anopheles gambiae *s.s., the principal vector of human malaria in sub-Saharan Africa, belongs to a complex including at least six other sibling species (*A. arabiensis, A. melas, A. merus, A. quadriannulatus *A and B and *A. bwambae*) that are morphologically indistinguishable, but remarkably distinct in their ecological adaptations, fixed chromosomal inversions, behaviours and role in malaria transmission [16,17]. Recent studies have revealed genes potentially involved in *A. gambiae *s.s. post-mating physiological and behavioral responses [18-20], thus opening perspectives to investigate their differentiation among the species of the *A. gambiae *complex [21]. Males of *A. gambiae *s.s. transfer their male accessory gland (MAG) products into the female atrium during mating as a solid mass, the "mating plug", that is digested in the space of 24-48 hours post-mating. In *A. gambiae *s.s., transfer of the mating plug plays an important role in reproduction, as females mated to males impaired in mating plug formation are not capable of storing sperm in their spermathecae [20]. A number of MAG proteins have been identified in *Anopheles *that are orthologs of *Drosophila *Acps [18-20]. A possible role for these male proteins in inducing female post-mating responses such as ovulation, oviposition and life-long mating refractoriness has been postulated [22,23]. Among the Acps identified in *A. gambiae *s.s., two small paralog genes, annotated on the *A. gambiae *genome (PEST strain, AgamP ver. 3.6) as AGAP009369 and AGAP009370 (86% of identity at the nucleotide level), were identified in the so-called *A. gambiae *male "fertilization island" located on chromosome 3 [18]. These genes lie in division 34A of the right arm of chromosome 3, and are close to the gene encoding the glutamine-rich protein "Plugin" (AGAP009368), the most abundant component of the mating plug. Plugin is the major substrate for the male-specific transglutaminase enzyme responsible for the coagulation of the liquid MAG secretions [20]. AGAP009369 and AGAP009370 proteins have been postulated to have a role in fertility and in female post-mating responses, based on their homology with *Drosophila Acp53Ea *protein, which has a role in sperm competitive ability and/or a hormonal activity [18,20]. Because of a lack of knowledge on their specific functions, we decided to assign provisional gene names to these two Acps by adopting the nomenclature commonly used for *Drosophila *Acps. Hence we used names to summarize the species- and tissue-specificity, chromosomal location and paralogy of these genes. Then, for the purpose of this work, AGAP009369 and AGAP009370 were provisionally renamed *AgAcp34A-1 *and *AgAcp34A-2*, respectively.

We present data on the genetic differentiation and expression of these male-expressed genes among the species of the *A. gambiae *complex and report the presence of a previously unannotated gene duplicate. The results also highlight the existence of species-specific products that might be indicative of unique lineage-specific functions of these proteins.

## Methods

### Field collected samples

**Figure 1:**
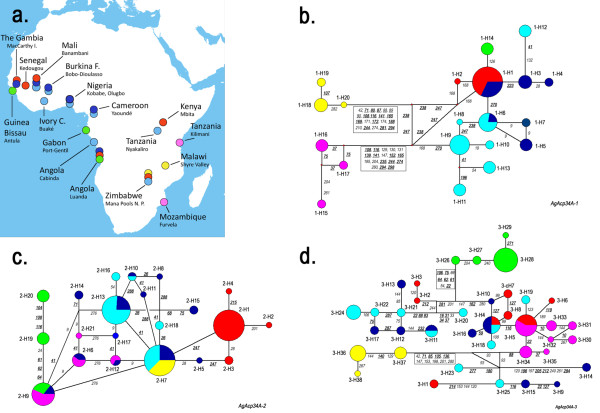
**Geographic distribution of *A. gambiae *s.l. samples and median-joining networks of coding haplotypes**. Species- and form-specific colours in the geographic map (a) and networks (b, c, d) are as follows: *A. gambiae *M-form: dark blue, *A. gambiae *S-form: light blue, *A. arabiensis*: red, *A. merus*: violet, *A. melas*: green, *A. quadriannulatus*: yellow. Synonymous mutations are in italics, nonsynonymous mutations are in bold-italics. The size of the circles in the networks is proportional to the haplotype frequencies. Numbering of mutations follows the complete coding sequence of each gene: *AgAcp34A-1 *(b), *AgAcp34A-2 *(c) and *AgAcp34A-3 *(d).

The study was carried out on a total of ~50-65 individuals belonging to five species of the *A. gambiae *complex and collected in several localities along their geographical distribution (Figure 1a). Samples of both incipient species within *A. gambiae *s.s. - namely the M and S molecular forms [24] - were also considered in our study. We selected a large sample from an extended geographical scale in order to more efficiently distinguish fixed differences among species from polymorphisms present in one or more species. *A. gambiae *s.s. M- and S-form adults were collected between 1998 and 2008 in 10 African countries (Angola, Burkina Faso, Cameroon, Ivory Coast, Mali, Nigeria, Senegal, Tanzania, The Gambia, Zimbabwe), *A. arabiensis *from 6 countries (Senegal, The Gambia, Mali, Angola, Zimbabwe and Kenya), *A. melas *from Angola, Gabon and Guinea Bissau, *A. quadriannulatus *A from Zimbabwe and Malawi and *A. merus *from Mozambique and Tanzania.

Genomic DNA was extracted from the head and thorax of each mosquito using standard procedures and specimens were identified to species and molecular forms using both PCR-RFLP [25] and *SINE200 *methods [26].

### PCR amplification and sequencing

**Figure 2:**
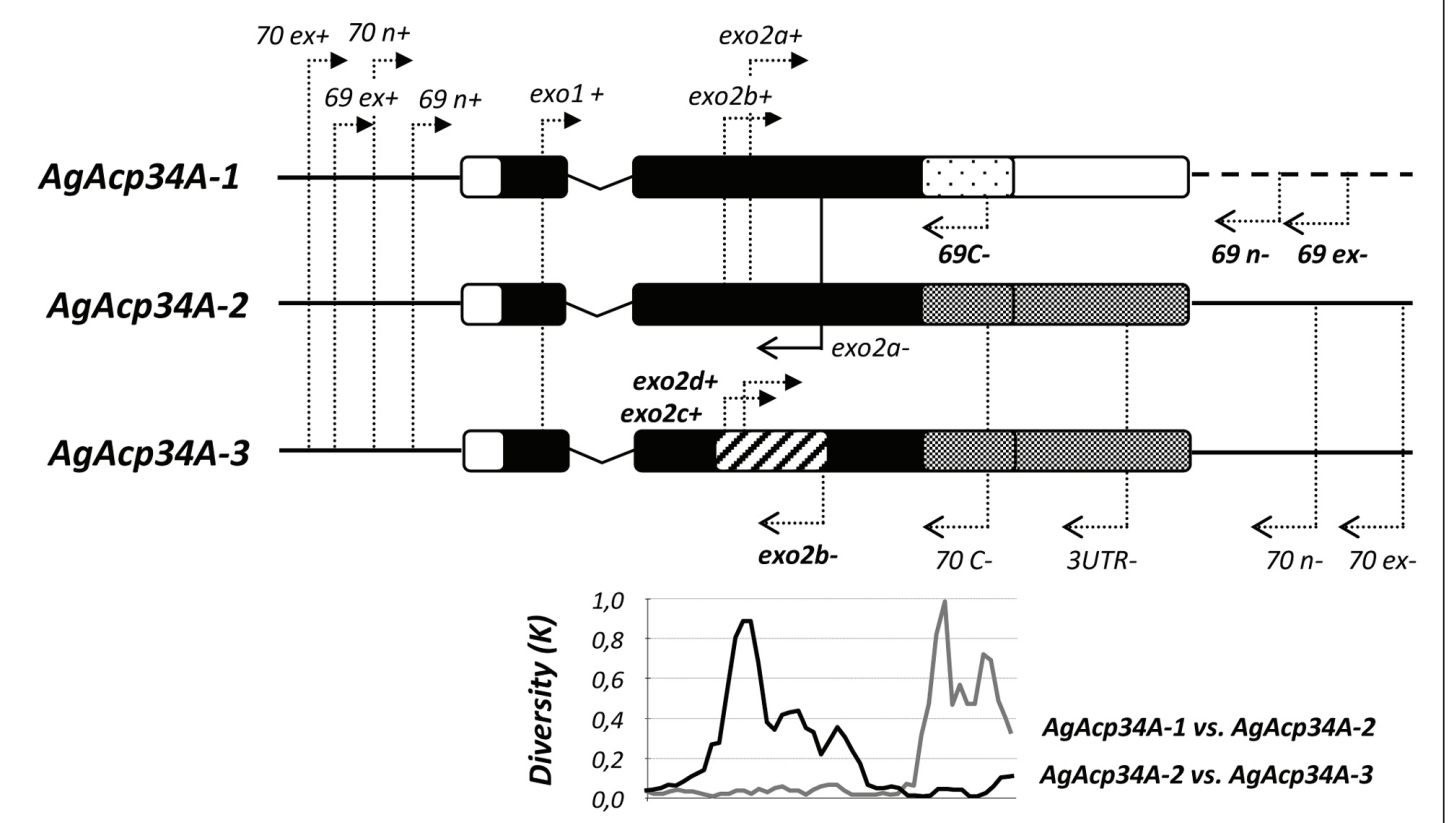
**Schematic maps of genes, primers and paralog diversity**. The gene structure of each male-expressed paralog is reported (and drawn to scale): bars indicate exons, the 'V' shaped line represents the intron, the thick lines on both sides (dashed on the right side for *AgAcp34A-1*) represent 5' and 3' flanking regions. Arrows indicate the positions of primers reported in Table 1. Diversity at nonsynymous sites (K) among paralogs (bottom part) was computed in a sliding window of 100 bp. Portions of genes showing more than ~20% of nonsynonymous diversity among paralogs are represented with different shading schemes.

Multiple primer pairs were designed using Gene Runner 3.05 (Hastings Software, Inc.) and the *A. gambiae *s.s. genome (PEST strain, AgamP3.6) as a reference, to specifically amplify and sequence each paralog gene in a single amplicon or in two overlapping fragments (Table 1, Figure 2). Due to the high similarity among the three paralogs (Figure 2), a nested PCR protocol was needed in most cases to ensure the specific amplification of the targeted gene. The whole sequence of *AgAcp34A-1 *(including 5'-3' UTRs) was obtained for all specimens in a single 632 bp amplicon using a nested PCR approach with reverse primers placed on the specific portion of its 3' flanking region (Figure 2). Similarly, the entire sequence of *AgAcp34A-2 *(including 5'-3' UTRs, 685 bp) was initially obtained from 34 specimens of *A. gambiae *and *A. arabiensis *using specific primer pairs with the reverse primer placed on its 3' flanking region, whereas a slightly different "variant" of *AgAcp34A-2 *was initially amplified with the same protocol from 27 specimens of *A. melas, A. merus *and *A. quadriannulatus*. However, both *AgAcp34A-2 *"variants" were then amplified in all *A. gambiae *complex specimens and later isolated using variant-specific primers, thus providing evidence for the presence of a duplicated gene of *AgAcp34A-2*, that we have named *AgAcp34A-3*. This produced a 100% "permanent heterozygosity" in our samples, as a consequence of the presence of fixed cismorphisms (i.e. fixed differences between paralogous sequences) [27]. Various PCR and sequencing strategies were then designed to obtain both copy-specific fragments from all *A. gambiae *s.l. specimens (Table 1, Figure 2).

**Table 1:** Primer list and amplification/sequencing strategies

PRIMER LIST				
**Gene**	**Primer**	**Forward primer (5'-3')**	**Reverse primer (5'-3')**	**(bp)**

*AgAcp34A-1*	69 ex	ATTGAACGAGCACCACCGC	ACAATGCAGAACCTTCGAC	735
*AgAcp34A-1*	69 n	ACGCCAGGCTTGTACTCTC	CACTTATAAACTAGCTACC	632
*AgAcp34A-2/3*	70 ex	ATTCCCGTAACTATCTTGC	TATAACTCACGGGCGATTC	793
*AgAcp34A-2/3*	70 n	AATGTGTCCTTTCTGAACC	CTGCCCAATTAACCAATAG	685
*AgAcp34A-1/2/3*	exo1 (+)	TCGCCCTAGTGGCTGTTG	-	-
*AgAcp34A-2/3*	3UTR (-)	-	TCGTCCATTCCCATCGCAG	-
*AgAcp34A-1/2*	exo2a (+)	TAGATAACAGACAGTTACC	-	-
*AgAcp34A-1/2*	exo2b (+)	ATGCCCATAAATACTTTAG	-	-
*AgAcp34A-1/2*	exo2a (-)	-	GTCAACAAGCCCTACAAGA	-
*AgAcp34A-3*	exo2c (+)	ATACCCAAMCTGCCTATGC	-	-
*AgAcp34A-3*	exo2d (+)	CTATGCGCCGGCAGGTTTC	-	-
*AgAcp34A-3*	exo2b (-)	-	ATTTTAGAAACCTGCCGGC	-
*AgAcp34A-1*	69 C (-)	-	TCTATAGAYAGTATCTACG	-
*AgAcp34A-2/3*	70 C (-)	-	AAATTGTTCATTGAGAGTC	-

**PCR STRATEGIES**				

**Nested PCR strategy to selectively amplify *AgAcp34A-2 *and *AgAcp34A-3***^**a**^				

**Gene**	**Portion**	**Forward primer (5'-3')**	**Reverse primer (5'-3')**	**(bp)**

*AgAcp34A-2*	N terminal	70 n (+)*	exo2a (-)	326
	C terminal	exo2a (+)	3UTR (-)*	276
*AgAcp34A-3*	N terminal	70 n (+)*	exo2b (-)	296
	C terminal	exo2c (+)	3UTR (-)*	289

**Nested RT-PCR strategy to selectively amplify transcripts (cDNA)**				

**Gene**	**Round**	**Forward primer (5'-3')**	**Reverse primer (5'-3')**	**(bp)**

*AgAcp34A-1*	1° round	exo1 (+)	69 C (-)	260
	2° round	exo2a (+)	69 C (-)	170
*AgAcp34A-2*	1° round	exo1 (+)	70 C (-)	266
	2° round	exo2a (+)	70 c (-)	176
*AgAcp34A-3*	1° round	exo1 (+)	70 C (-)	263
	2° round	exo2c (+)	70 C (-)	189

^a ^1° round using common external primers: 70 ex (+)/(-); * sequencing primers

PCR reactions were carried out in a 25 μl reaction which contained 1 pmol of each primer, 0.2 mM of each dNTP, 1.5 mM MgCl_2_, 2.5 U Bioline Taq polymerase, and 0.5 μl of template DNA extracted from a single mosquito. Thermocycler conditions were 94°C for 10 min followed by 35 cycles of 94°C for 30 s, 50-54°C for 30 s and 72°C for 1 min., with a final extension at 72°C for 10 min. For nested PCR, fragments obtained with a specific set of primers were diluted 1:100 and used as a template for subsequent PCR using internal primers.

The resulting products were analysed on 1% agarose gels stained with ethidium bromide. The PCR products were purified using the SureClean Kit (Bioline) and sequenced on both strands at the BMR Genomics s.r.l (Padua, Italy). All sequences were deposited in GenBank under Accession Nos. JN694584-JN694752.

### BLAST searches in the *A. gambiae *genome

The program Consensus (threshold set = 50; http://coot.embl.de/Alignment//consensus.html) was used to generate consensus sequences from the alignments obtained from *A. gambiae *s.s. specimens for either *AgAcp34A-2 *and *AgAcp34A-3*. We then used the consensus sequences as a query and conducted BLASTn searches by setting the e-value cutoff = e-10 and a BLOSUM62 default scoring matrix in http://agambiae.vectorbase.org/Tools/BLAST/ on: i) the *A. gambiae *genome assembly (AgamP3.6), ii) the *A. gambiae *PEST strain trace reads, iii) M (Mali NIH strain) and S (Pimperena strain) contigs, scaffold and trace reads. Trace read (and mate pair) chromatograms were recovered from http://www.ncbi.nlm.nih.gov/Traces.

### Fluorescence *in situ *hybridization (FISH)

To determine the chromosomal localization of *AgAcp34A-1, AgAcp34A-2 *and *AgAcp34A-3*, we designed probes that were 632 bp- and 685 bp-long. A nested PCR approach was used to amplify these probes. A first round of PCR with the primer pair "69 ex" (Table 1) produced a 735 bp fragment of *AgAcp34A-1*. The second round of PCR with the primer pair "69 n" (Table 1) specifically amplified a 632 bp fragment of *AgAcp34A-1*. For amplification of *AgAcp34A-2 *and *AgAcp34A-3 *we used the primer pair "70 ex" (Table 1) that produced a 793 bp fragment, followed by a nested PCR with the primer pair "70 n" (Table 1) that produced a 685 bp fragment specific to both *AgAcp34A-2 *and *AgAcp34A-3*, but not to *AgAcp34A-1*. The genomic DNA of single *A. gambiae *SUA mosquitoes was extracted with the Wizard SV Genomic Purification System (Promega Corporation, Madison, WI, USA) and used as a template for PCR. PCR products were gel purified using the Geneclean kit (Qbiogene, Inc., Irvine, CA). The DNA was labeled with Cy3-AP3-dUTP and Cy3-AP3-dUTP (GE Healthcare UK Ltd., Buckinghamshire, England) using Random Primers DNA Labeling System (Invitrogen Corporation, Carlsbad, CA, USA).

Chromosomal preparations were made from the ovaries of half-gravid females of the SUA strain of *A. gambiae*, the OPHANSI strain of *A. merus*, and the DONGOLA strain of *A. arabiensis*. The *in situ *hybridization procedure was performed as previously described [28]. DNA probes were hybridized to the chromosomes at 39^°^C overnight in hybridization solution (Invitrogen Corporation, Carlsbad, CA, USA). Then the chromosomes were washed in 0.2XSSC (Saline-Sodium Citrate: 0.03 M Sodium Chloride, 0.003 M Sodium Citrate), counterstained with YOYO-1, and mounted in DABCO. Fluorescent signals were detected and recorded using a Zeiss LSM 510 Laser Scanning Microscope (Carl Zeiss MicroImaging, Inc., Thornwood, NY, USA). The locations of signals were determined using a standard cytogenetic photo map of *A. gambiae *[29].

Confocal images were processed using ImageJ and Adobe Photoshop software by splitting color channels from the initial RGB image into separate images as described elsewhere [30]. Each channel image was converted into the monochrome image by using a "Channel mixer" and then inverted. The inverted monochrome image was adjusted by using a "Curves" tool until the background was removed and each chromosome of the spread became fuzzy-edged. The reduction of noise was achieved by blurring of each pixel with the Gaussian blur filter tool. The quality of the image was improved by additional application of the "Curves" and/or subtraction of the "Relative white". Finally, green channel image with chromosomes was merged with monochrome image of FISH signals. Processing yielded contrasted, inverted, grayscale images with color labels, which are more suitable for mapping.

### Sequence data analysis

All sequences were edited and assembled using the Staden Package ver. 2003.1.6 [31]. When sequences yielded a composite chromatogram at two or more sites, an indirect approach of haplotype estimation was performed using the PHASE algorithm - a Bayesian approach based on *a priori *prediction from the coalescent theory - implemented in DNAsp v5 [32]. After removing non-coding portions, codon alignments were recovered from each protein using MAFFT ver. 5 [33].

Basic analyses of genetic polymorphisms and neutrality tests were computed at the species level using DnaSP v5 [32]. The statistical significance of neutrality tests was obtained using 10000 coalescent simulations in DnaSP v5. The average pairwise differences among species of the *A. gambiae *complex for each sequence dataset were calculated using Arlequin v3.11 [34].

A median-joining network [35] was built for each gene with the program NETWORK version 4.510 http://www.fluxus-engineering.com/ to display relationships among the coding haplotype sequences.

A scan for recombination at each locus was performed using the seven methods implemented in the RDP3 software [36]. Gene conversion was detected using GENECONV [37]http://www.math.wustl.edu/~sawyer/geneconv/ within alignments of the obtained haplotypes by all paralogs. The statistical significance of highly similar tracts (representing conversion events) identified by GENECONV was scored by the permutation tests.

### Gene expression

Tissues from MAG of 4- to 5-day-old virgin adults were dissected in 1 × PBS solution from specimens of SUA strain of *A. gambiae*, of OPHANSI strain of *A. merus*, and of DONGOLA strain of *A. arabiensis*, and total RNA was extracted from MAG, as well as from the rest of male body (carcass) and whole adult female tissues using TRIZOL Reagent (Invitrogen), according to the manufacturer's instructions. For each species, RNA was extracted from three replicates obtained for each tissue (MAG, male carcasses and whole adult female). Purified RNA was treated with RNase-free DNase I (Invitrogen). Reverse transcription reactions were performed in 20 uL volumes by using 4 uL of 5 × First Strand Buffer, 2 uL of 0.1 M DTT, 1 uL of 10 mM dNTPs, 150 ng of random hexamers, 1 ul of RNaseOUT recombinant ribonuclease inhibitor, and 200 Units M-MLV Reverse Transcriptase (all reagents from Invitrogen). Resultant cDNA was diluted to 10 ng/ul and and 1 ul was used as a template for subsequent nested RT-PCR protocols. Nested RT-PCR protocols (primer couples are reported in Table 1, nested PCR conditions were the same as for sequencing) were optimized to reliably distinguish paralog-specific transcripts and to determine their presence even at low expression level, although preventing the assessment of their relative abundance. Nested RT-PCRs were replicated at least three times for each gene and products were sequenced on both strands to confirm their identity. The ribosomal gene S7 was used as a positive control for all cDNA samples to prevent genomic DNA contamination (primer used for rpS7 amplification: Ag rpS7 fw 5'-GGCGATCATCATCTACGTGC-3', Ag rpS7 rev 5'-GTAGCTGCTGCAAACTTCGG-3').

### Immunostaining analysis of male accessory glands and mating plug

Mating plugs dissected from recently mated *A. gambiae *females, and MAG dissected from *A. gambiae, A. arabiensis*, and *A. merus *virgin 4- to 5-day-old males were fixed in PBS 4% formaldehyde solution. After washing in PBS, the samples were bleached with 2% hydrogen peroxide to reduce autofluorescence, washed in PBS and then blocked and permeabilized in PBS with 1% BSA and 0.1% Triton X-100. Then the samples were incubated with 1.38 μg/ml anti-AGAP009370-like (i.e. *AgAcp34A-2*/3) in blocking buffer, washed, stained with anti-Rabbit Alexa-488 (Invitrogen) at a 1:1000 dilution followed by a 1:250 dilution of Phalloidin-Alexa-546 (Invitrogen) to stain actin. Tissues were then mounted in DAPI-containing Vectashield medium (Vector Laboratories, Inc.) and visualized using a Leica SP5 inverted confocal microscope or a Zeiss Axio Observer inverted fluorescent microscope with apotome. Affinity-purified polyclonal antibodies against the AGAP009370-like protein (i.e. *AgAcp34A-2/3*) were raised in rabbit against a peptide epitope (CLPPFAKTLNEQFGQ; common to both *AgAcp34A-2 *and *AgAcp34A-3*) by a commercial supplier (Gen-Script Corp., Piscataway, NJ).

### Western blot

MAG, rest of male tissues and mating plugs of *A. gambiae *s.s. were homogenized in 15 μl of Extraction Buffer (Tris-HCl 25 mM pH 7.4, NaCl 150 mM, EDTA 10 mM pH 8.0, protease inhibitor cocktail 1 × (CompleteMini, Roche), SDS 0.1%, Triton X-100 1%, Nonidet P-40 1%). After 15 minutes of incubation on ice, samples were centrifuged at 13,000 rpm for 15 minutes at 4°C. Protein concentration in the supernatant was then quantified using Bradford method (Bio-Rad Laboratories). NuPAGE LDS Sample Buffer (Invitrogen) and dithiotreitol at 100 mM final concentration were then added to the protein extracts. Samples were then heated at 70°C for 10 minutes and applied to precast NuPAGE (Invitrogen) gels according to the manufacturer's instructions. Proteins were transferred to a nitrocellulose membrane using the XCell II Blot module (Invitrogen). Blots were immunostained using standard protocols with the following primary antibody titres: anti-AGAP009370-like: 0.46 μg/ml; anti-β-actin (1:1000 dilution clone ZCA34 Invitrogen). HRPconjugated secondary antibodies (Santa Cruz Biotechnologies: sc-2030 and sc-2314) were used at a dilution of 1:10000. Bands were visualized using ECL Western Blotting detection reagents (GE Heatlhcare) on an Fusion FX7 imaging system (Vilbert Lonrmat).

## Results

### Identification of *AgAcp34A-3*, a novel gene duplicate of *AgAcp34A-2*

Reproducible "permanent heterozygosity" in sequence data - initially obtained from most of the analyzed individuals of the *A. gambiae *complex species using primers placed on flanking regions of *AgAcp34A-2 *(Table 1) - provided a first clue of the putative presence of a genomic duplication. The existence of an additional duplicate, *AgAcp34A-3*, was then confirmed by obtaining specific amplification of both paralogs in all individuals and by observing for each gene duplicate paralog-specific heterozygous SNPs (double peaks) in chromatograms. Blast searches on the annotated *A. gambiae *assembly (AgamP3.6) failed to retrieve *AgAcp34A-3 *(9 hits on the genome corresponding to the sequences of the genes annotated as AGAP009369, AGAP009370 or AGAP0012706, but not to *AgAcp34A-3*; E-value = 1e^-55 ^< E < 7e^-17^), thus indicating that this gene is not mapped on the *A. gambiae *genome. However, when we surveyed the trace read repositories of the whole *A. gambiae *s.s. genome for the occurrence of these two copies, we found 10 trace reads that unambigously matched the *AgAcp34A-3 *reference sequence: 1 in "PEST strain" (trace read ID: 117500516), 5 in M-form "Mali-NIH strain" (trace read ID: 1543277868, 1537713761, 1527392852, 1517773106, 1524910694), and 4 in S-form "Pimperena strain" (trace read ID: 1443434889, 1430388232, 1474892160, 1475087970). On the contrary, when *AgAcp34A-2 *sequence was used as a query, 10 and 2 trace reads stored in the *A. gambiae *PEST strain and S-form database, respectively, perfectly matched the reference sequence, whereas no match was found in the M-form repository. Although the two "variants" are stored in both PEST and S-form strain trace archives, only *AgAcp34A-2 *(i.e. AGAP009370) is annotated in the latest version of the *A. gambiae *genome map, whereas only *AgAcp34A-3 *is assembled in the S-form contig ABKQ01012658.1 (scaffold EQ099715.1). Interestingly, in both M- (ABKP02025712.1, scaffold EQ090190.1) and S-form contigs (ABKQ01012658.1, scaffold EQ099715.1) *AgAcp34A-3 *is placed exactly in the same locus occupied by *AgAcp34A-2 *in the PEST genome. It is worth noting that the flanking regions of both paralogs are virtually undistinguishable, at least based on the quality cut-off (> 20) of trace files. We also retrieved "mate pairs" (i.e. the reads obtained by sequencing the same clone on the opposite direction) of recovered trace files to gain more information about the position of the two genes in the genome. We found that when mate pairs were readable, they did not always map between AGAP009368 (3R:31814166-31814685) and AGAP009371 (3R:31822600-31823056), but were rather placed within the 3R_hap_15 region (31748711-31776836) that represents an alternative assembly of a larger portion of chromosome 3R (3R:31748711-31785547). This led us to suspect the presence of a large duplicated genomic region containing *AgAcp34A-3 *possibly affecting the annotation of this gene.

**Figure 3:**
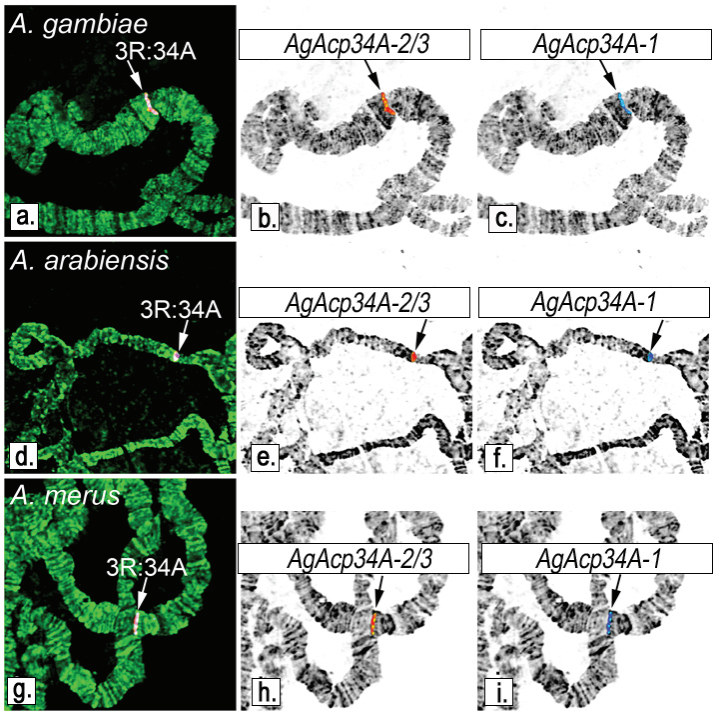
**FISH mapping of the genes on polytene chromosome of *A. gambiae, A. arabiensis *and *A. merus***. FISH was performed with a probe specific to *AgAcp34A-1 *and a probe common to *AgAcp34A-2 *and *AgAcp34A-3 *on polytene chromosomes of *A. gambiae *(top panel), *A. arabiensis *(middle panel), and *A. merus *(bottom panel). Overlapping red and blue signals on chromosomes counterstained with YOYO-1 are shown on merged images (a, d, g). The *AgAcp34A-2*/*3 *probe is labeled with Cy3-AP3-dUTP (red signal in b, e, h). The *AgAcp34A-1 *probe is labeled with Cy5-AP3-dUTP (blue signal in c, f, i). Arrows indicate the site of localizations of the blue and red signals in subdivision 34A of the 3R arm.

FISH performed on *A. gambiae, A. arabiensis *and *A. merus *chromosomes using two probes binding to *AgAcp34A-1 *and to the common sequence of *AgAcp34A-2 *and *AgAcp34A-3*, respectively, each returned a single signal in subdivision 34A of the 3R-chromosome arm (Figure 3).

Overall, these results suggest that *AgAcp34A-3 *is located in the same chromosomal division as the other two genes in *A. gambiae *s.s. and in all examined species of the *A. gambiae *complex, indicating that a tandem duplication occurred in this genomic region.

### Polymorphism and divergence in coding sequences

A summary of intra-specific sequence variation in the *A. gambiae *complex is shown in Table 2.

**Table 2:** Summary statistics of polymorphisms and neutrality test for *AgAcp34A-1, AgAcp34A-2 *and *AgAcp34A-3 *sequence data

Gene	Species/form	N	n	H	Hd	S	π	θ	S	NS	**π**_**s**_	**π**_**a**_	*D*
***AgAcp34A-1***	***A. gambiae***	28	56	12	0.90	11	0.009	0.008	6	6	0.009	0.010	0.510
	***A. gambiae *M**	12	24	6	0.80	4	0.005	0.004	3	2	0.008	0.004	1.030
	***A. gambiae *S**	16	32	7	0.83	9	0.009	0.007	3	6	0.008	0.009	0.615
	***A. arabiensis***	10	20	2	0.10	1	0.000	0.001	1	0	0.001	0.000	-1.164
	***A. melas***	2	4	1	0.00	0	0.000	0.000	0	0	0.000	0.000	n.a.
	***A. quadriannulatus***	4	8	3	0.61	2	0.002	0.003	1	1	0.003	0.002	-0.448
	***A. merus***	4	8	3	0.71	4	0.006	0.005	2	2	0.012	0.004	0.889
	
	**total**	48	96	20	0.89	46	0.029	0.031					
													
***AgAcp34A-2***	***A. gambiae***	30	60	14	0.77	10	0.005	0.007	4	6	0.006	0.004	-0.968
	***A. gambiae *M**	13	26	11	0.87	9	0.007	0.008	3	6	0.010	0.006	-0.442
	***A. gambiae *S**	17	34	6	0.67	5	0.003	0.004	2	3	0.003	0.003	-0.817
	***A. arabiensis***	16	32	4	0.24	3	0.001	0.003	1	2	0.002	0.001	-1.548
	***A. melas***	6	12	3	0.73	7	0.012	0.008	1	6	0.007	0.013	1.816
	***A. quadriannulatus***	6	12	1	0.00	0	0.000	0.000	0	0	0.000	0.000	n.a.
	***A. merus***	7	14	4	0.65	3	0.003	0.003	2	1	0.012	0.001	-0.030
	
	**total**	65	130	21	0.86	20	0.008	0.013					
													
***AgAcp34A-3***	***A. gambiae***	22	44	18	0.95	36	0.029	0.028	11	20	0.044	0.025	0.088
	***A. gambiae *M**	10	20	10	0.93	25	0.029	0.024	8	17	0.043	0.024	0.711
	***A. gambiae *S**	12	24	10	0.91	26	0.029	0.024	8	13	0.043	0.022	0.766
	***A. arabiensis***	8	16	8	0.88	20	0.017	0.021	9	11	0.033	0.013	-0.641
	***A. melas***	10	20	4	0.44	3	0.002	0.003	2	1	0.008	0.000	-0.693
	***A. quadriannulatus***	6	12	3	0.59	4	0.006	0.005	3	1	0.017	0.002	0.788
	***A. merus***	10	20	7	0.83	5	0.005	0.005	2	3	0.009	0.004	0.140
	
	**total**	56	112	38	0.96	58	0.038	0.038					
													

N = n° individuals; n = n° alleles; H = n° haplotypes; S = n° segregating sites; Hd = haplotype diversity; π = nucleotide diversity; at synonymous (π_s_) and (π_a_) nonsynonymous sites; θ = Watterson estimate of theta; S and NS = total number of silent and replacement mutations; D = Tajima D value; p = statistical significance using confidence levels provided by the coalescent (DNAsp 5.0) of Tajima D values (none significant)

For *AgAcp34A-1 *a 300-bp coding sequence was obtained from 48 individuals. On average, 46 segregating sites were found (15% of the total number of nucleotide sites) and 26 out 100 (26%) amino acid positions were variable. The average nucleotide diversity (π) was 0.029 and 20 haplotypes were identified (out of 96 alleles). The highest haplotype diversity (Hd) was found in *A. gambiae *s.s. M- (0.80) and S- (0.83) forms. In general, low π values were scored within *A. gambiae *species/forms (0.000-0.009), both at synonymous (π_s _= 0.000-0.012) and nonsynonymous (π_a _= 0.000-0.010) sites.

For *AgAcp34A-2 *a 294-bp coding sequence was obtained from 65 individuals. On average, 20 segregating sites were found (7% of the total number of nucleotide sites) and 14 out 98 (14%) amino acid positions were variable. The average π was 0.008 and 21 haplotypes were identified (out 130 alleles). The highest Hd was found in M- (0.87) and S- (0.67) forms. Low π values were scored within *A. gambiae *species/forms (0.000-0.012), both at synonymous (π_s _= 0.000-0.012) and nonsynonymous (π_a _= 0.000-0.013) sites.

For *AgAcp34A-3 *a 291 bp coding sequence was obtained from 56 individuals. On average, 58 segregating sites were found (20% of the total number of nucleotide sites) and 36 out of 97 (37%) amino acid position were variable. The average π was 0.038 and 38 haplotypes were identified (out of 112 alleles). The highest Hd was found in M- (0.93) and S- (0.91) forms. Notable high π values were scored within *A. gambiae *s.s. molecular forms (0.029) and *A. arabiensis *(0.017), at both synonymous (M- and S-forms π_s _= 0.043, *A. arabiensis *π_s _= 0.033) and nonsynonymous (M-form π_a _= 0.022, S-form π_a _= 0.024, *A. arabiensis *= 0.013) sites.

At species level, the Tajima test [38] did not detect any significant deviation from neutral expectation at coding sites of all genes. However, for *AgAcp34A-2 *Tajima D statistics were negative in *A. gambiae *and *A. arabiensis*, thus indicating an excess of rare or recent mutations that could be due to a recent demographic expansion or to purifying selection. A high - although nonsignificant - positive Tajima's D value was obtained for *A. melas*, indicating low levels of both low and high frequency polymorphisms, possibly because of a decrease in population size and/or balancing selection.

Finally, we observed low levels of sequence divergence between *A. gambiae *molecular forms for all three genes (*AgAcp34A-1 *= 0.005, *AgAcp34A-2 *= 0.012, *AgAcp34A-3 *= 0.036). The average pairwise sequence differences ranged from 0.003 (*A. gambiae*-M *vs. A. arabiensis*) to 0.124 (*A. merus vs. A. quadriannulatus*) for *AgAcp34A-1*, from 0.003 (*A. gambiae*-S *vs. A. quadriannulatus*) to 0.020 (*A. arabiensis vs. A. melas*) for *AgAcp34A-2*, and from 0.015 (*A. arabiensis vs. A. merus*) to 0.122 (*A. melas vs. A. quadriannulatus*) for *AgAcp34A-3*.

### Network analyses of coding haplotypes

**Figure 4:**
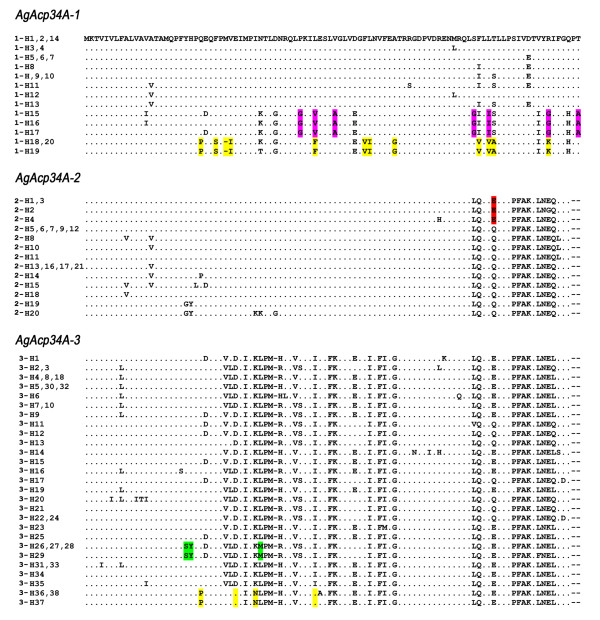
**Alignments of *A. gambiae *species proteins predicted from haplotypes obtained for each gene**. Haplotypes coding for the same protein (and named as in the median-joining networks in Figure 1) are reported on the left. A dot indicates the same residue as in the first sequence, dashes indicate deletions. Species-specific fixed amino acid substitutions are coloured as follows: *A. arabiensis*: red, *A. merus*: violet, *A. melas*: green, *A. quadriannulatus*: yellow.

The median-joining networks based on the *AgAcp34A-1 *coding sequence showed a clear separation of *A. quadriannulatus *and *A. merus *from the other species of the complex (Figure 1b). In fact, haplotypes 1-H18, 1-H19 and 1-H20 were unique to *A. quadriannulatus *(which is distinguished from all other species by 11 fixed species-specific replacements and 1 amino acid deletion, Figure 4), and separated from all other haplotypes by at least 18 nonsynonymous mutations (Figure 1b). Similarly, haplotypes 1-H15, 1-H16 and 1-H17 were unique to *A. merus *(which is distinct from all other species by 7 fixed species-specific replacements, Figure 4) and distant for at least 13 nonsynoymous substitutions from all other haplotypes. With the only exception of one allele from Senegal population (1-H2), all other *A. arabiensis *sequences were grouped in haplotype 1-H1, which is also shared with 37% of *A. gambiae *M-form alleles (Figure 1b) and closely related to the *A. melas *specific haplotype 1-H14 (i.e. separated by a single synonymous substitutions at position 126). Note that *A. arabiensis *and *A. melas *share the same protein sequence (Figure 4). A slight separation between M- and S-forms can be also observed: i) haplotypes 1-H8, 1-H9, 1-H10, 1-H11 and 1-H13 are closely related and S-form specific (owning 1 to 4 S-form private nonsynonymous substitutions, Figure 1a); ii) haplotype 1-H12 is S-form specific, although related to M-form specific haplotypes 1-H3 and 1-H4; iii) haplotype 1-H6 is shared by 21% and 8% of S- and M-form alleles, respectively, and connected to M-form specific haplotypes 1-H5 and 1-H7.

The network based on the *AgAcp34A-2 *haplotypes (Figure 1c) showed nearly the same number of haplotypes if compared to the *AgAcp34A-1 *network (21 versus 20), although with a less clear separation between *A. gambiae *species/forms. An interesting exception is represented by haplotype 2-H1 and its peripheral closely linked haplotypes 2-H2, 2-H3 and 2-H4, which are unique to *A. arabiensis*. Notably, this separation is due to a single and fixed nonsynonymous mutation at position 247 in the C-terminal portion that causes the change of a glutamine (Q) to a glutamic acid (E) in *A. arabiensis *(Figure 4). Two other high frequency haplotypes are shared by more than one taxon: 2-H7 which includes all *A. quadriannulatus *and 31% - 37% of M-and S-form alleles respectively, and 2-H13 including 19% of M-form and 47% of S- form alleles. *A. merus *shares haplotypes with the M-form (2-H6 and 2-H12) and *A. melas *(2-H9), the latter being connected with two *A. melas *specific and separated haplotypes (2-H19 and 2-H20).

A more complex network with 38 haplotypes was reconstructed for *AgAcp34A-3 *(Figure 1d). In fact, most of the haplotypes were unique, although two of them were shared among some species (3-H4 and 3-H5) and one between M- and S-forms only (3-H11). Exclusive haplotypes of *A. gambiae *and *A. arabiensis *were interspersed but, in most cases, connected in the network without any geographic structuring. Haplotype 3-H5 is shared by *A. arabiensis *and *A. merus *and closely related to all other 6 *A. merus *specific haplotypes. Interestingly, we observed a clear separation of *A. quadriannulatus *and *A. melas *from the other species of the complex (Figure 1d): haplotypes 3-H36, 3-H37 and 3-H38 were unique to *A. quadriannulatus *(which is distinguishable from all other species by 4 fixed species-specific replacements, Figure 4) and haplotypes 3-H26, 3-H27, 3-H28, 3-H29 were unique to *A. melas *(bearing 3 fixed replacements unique for this species, Figure 4).

### Protein diversity, identity levels of paralogs and gene conversion

Figure 4 shows the alignment of protein types (associated to haplotypes in the networks) that are encoded by the three paralogs.

There were 13 protein sequences in our *AgAcp34A-1 *sample (Figure 4). The species-specific proteins of *A. merus *and *A. quadriannulatus *(encoded by haplotypes 1-H15 - 1-H20, see Figure 1b) differed by about 15% of residues from those of *A. gambiae, A. arabiensis *and *A. melas*, which differed from each other by only 1-4% of amino acid residues. Few amino acid differences (3.5%) were found among the 13 *AgAcp34A-2 *protein sequences (Figure 4), whereas a higher number of amino acid differences (6.9%) were scored among the 27 sequences found in our *AgAcp34A-3 *dataset (Figure 4). Overall, *AgAcp34A-2 *shared 83% and 78% of residues with *AgAcp34A-1 *and *AgAcp34A-3*, respectively. A relatively smaller number of residues (68%) were shared between *AgAcp34A-1 *and *AgAcp34A-3*. All proteins were very similar in their signal peptide portion (residues 1-16). *AgAcp34A-1 *was differentiated from *AgAcp34A-2 *and *AgAcp34A-3 *by 9 fixed replacements and 2 amino acid deletions at the C-terminal portion (residues 79-100). The C-terminal part of *AgAcp34A-2 *and *AgAcp34A-3 *was highly conserved, while these two copies differed by 14 fixed amino acid replacements and 1 residue deletion located at the N-terminal of the secreted peptide. Regions of complete identity shared among pairs of paralog haplotypes indicative of gene conversion events were identified: a 125-147 bp long gene conversion tract (from position 70 to position 195, 213 or 216) in the N-terminal portion between *AgAcp34A-1 *and *AgAcp34A-2 *and a 94-106 bp long converted fragments in the C-terminal portion (from position 194 to position 288 or 300) between *AgAcp34A-2 *and *AgAcp34A-3*. Finally, a putative ectopic recombination event (~40-60 bp long) involving the genetically more distant paralogs, *AgAcp34A-1 *and *AgAcp34A-3*, was detected and a 3' recombination breakpoint was identified at position 90 in the N-terminal portion.

### Expression and protein localization

We used a nested RT-PCR approach to analyze the expression of the three paralogs. This approach gives a clear picture of the presence/absence of transcripts in the analyzed tissues, because it allows detection of rare transcripts, although their relative amounts cannot be assessed. The three copies showed an identical expression pattern in the examined species (i.e. *A. gambiae, A. arabiensis *and *A. merus*). The genes were expressed in MAG (as well as in the rest of the body of adult males), while no expression could be detected in females (Additional file 1). Sequencing of RT-PCR products confirmed the identity of the three different transcripts.

**Figure 5:**
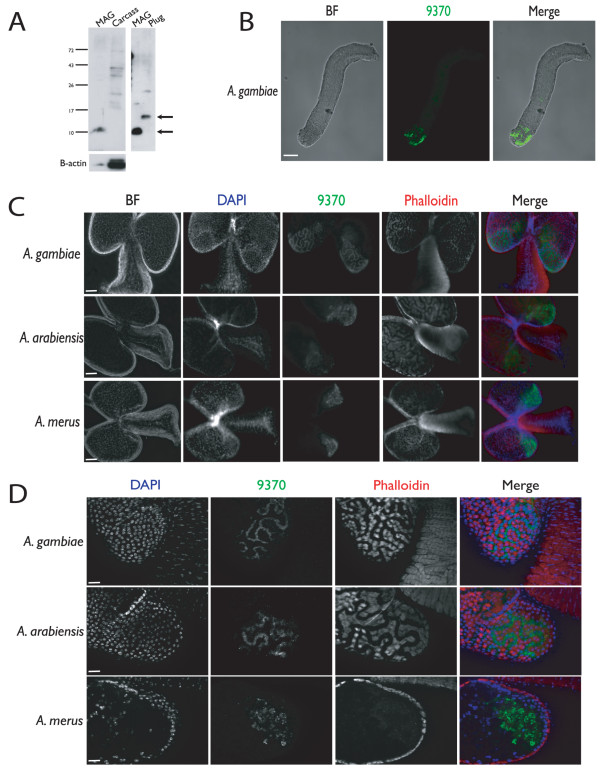
**Immunofluorescence and confocal analysis**. a) Western blot analysis of the AGAP009370-like protein (i.e. *AgAcp34A-2 *and/or *AgAcp34A-3 *gene products) using a polyclonal antibody raised against the C-terminal peptide fragment of the protein. AGAP009370-like is detected in the male accessory glands (MAG) and mating plug, but not in the carcass (rest of the body) of adult males. Arrows indicate the AGAP009370-like immunoreactive bands. Actin was used as a loading control. b) Confocal analysis of AGAP009370-like localization in the plug. The protein (green) is specifically localized in the posterior part. Scale bar: 44 μm. Fluorescent microscope analysis of MAG of virgin 3-day-old *A. gambiae, A. arabiensis *and *A. merus *males: c) AGAP009370-like (green) is primarily present in the posterior part of MAG (near the aedeagus) in all three species. Scale bar: 50 μm. d) The apotome analysis reveals that AGAP009370-like (green) is either present in channels formed by phalloidin-Alexa 546 (red)-labelled actin-rich muscle cells or in the lumen of the gland. The three species do not differ in this pattern. Here, channel localized protein is shown for *A. gambiae *and *A. arabiensis *and lumen presence for *A. merus*. Nuclei are labeled with DAPI (blue). Scale bar: 20 μm.

Western blot analyses of *A. gambiae *s.s. adult male tissues using polyclonal antibodies against the C-terminal of an AGAP009370-like protein (i.e. *AgAcp34A-2 *and/or *AgAcp34A-3*) gave a 10 kDa band in MAG protein extracts, which corresponds to the predicted molecular weight of this protein (Figure 5a). Specific bands were not present in the rest of male body (carcass) suggesting that MAG are the principal organs producing this protein. Furthermore, a 15 kDa band was detected in mating plug extracts (Figure 5a), confirming that the AGAP009370-like protein is transferred to females during copulation: we hypothesize that the higher molecular weight band observed for this protein in the mating plug extract, as compared to that predicted and observed in MAG, could be due to post-translational modifications of these male factors.

Immunofluorescence analysis was used to localize the AGAP009370-like protein in MAG and mating plugs dissected from recently mated females (Figure 5b, c, d). These experiments revealed that it was localized on the "posterior tip" of the mating plug in *A. gambiae *s.s., where the seminal secretions are in close proximity to the spermathecal duct (Figure 5b). In the MAG of all three species, the protein is localized in the posterior part of this organ (the portion near the aedeagus [20]) and accumulate in extracellular channels formed by the muscle layer surrounding the glands (Figure 5c). In some cases (as shown here for *A. merus *in Figure 5d) the protein is also visible in the gland lumen.

## Discussion

We analyzed an *A. gambiae *gene family in the "fertilization island" of chromosome arm 3R, where male-specific genes possibly involved in modulating female post-mating behavioural responses are located [18]. The following evidence strongly suggest the presence of a duplicate of *AgAcp34A-2 *gene, that we have named *AgAcp34A-3*: i) a consistent, reproducible permanent heterozygosity in sequence data obtained for *AgAcp34A-2 *in all specimens, and ii) the detection of SNPs (i.e. double peaks in chromatograms) in each paralog-specific sequence. Although trace reads of this novel duplicate are found in the repository of the *A. gambiae *genome (as well as in those of M- and S-forms), the annotation and localization of this copy on the genome map is probably hindered by the presence of a segmental duplication which complicates the assembly. In fact, our FISH assays suggest that this gene is placed in the same chromosomal division of *AgAcp34A-2 *in *A. gambiae, A. arabiensis *and *A. merus *(Figure 3) and that a tandem duplication likely occurred in this genomic region in a common ancestor of the *A. gambiae *complex. Note that, presently, two genes are reported as putative paralogs of *AgAcp34A-2 *in the *A. gambiae *genome: AGAP009369 (i.e. *AgAcp34A-1*, sharing 86% of identity) - mapping on the same chomosomal division as *AgAcp34A-2 *- and AGAP012706 (99% of identity with *AgAcp34A-2*), which is annotated on the artificial "unknown chromosome" containing unassigned scaffolds. However, the high level of identity between AGAP012706 and *AgAcp34A-2 *at both coding/non-coding and flanking regions, raises doubts on the actual existence of this additional paralog and suggests that this should rather be considered an alternative haplotype of *AgAcp34A-2 *[18].

Overall, the *AgAcp34A-2 *protein shares 83% and 78% of amino acid residues with *AgAcp34A-1 *and *AgAcp34A-3 *proteins, respectively. A relatively smaller number of residues (68%) are shared between *AgAcp34A-1 *and *AgAcp34A-3 *(see also Figure 4). Since the three paralogs are present in all analyzed species, it is likely that duplication events happened before the split of Afrotropical *A. gambiae *complex species from a common ancestor, and even before the divergence of the complex from the Asian malaria vector *A. stephensi*. In fact, we identified all three genes also in the latter species (data not shown) and *AgAcp34A-2 *can be found in its transcriptome [39]. Although it is difficult to determine with certainty when and how these duplications occurred, as well as the sequence of duplication events, it is likely that the most closely related copies (i.e. *AgAcp34A-1 *or *AgAcp34A-3*) could represent the direct descendant of an ancestral gene from which the other paralogs would have originated. A gene duplication of this ancestral gene could have given rise to a copy that, as a result of functional redundancy [15], would have been free to accumulate mutations along its coding and flanking regions. Subsequently, a gene conversion event between these two copies would have homogenized their N-terminal portions, as well as part of their 5' flanking regions. This would explain the finding of the high diversity observed mostly at the C-terminal and the 3' flanking region between *AgAcp34A-1 *and *AgAcp34A-2 *(Figure 4). A novel gene duplication event (possibly due to an unequal cross-over) would have then generated a third copy (the ancestor of *AgAcp34A-3*) from an AGAP009370-like ancestral gene. Again, this new duplicate would have accumulated mutations leading to fixation of several amino acid changes possibly under selective pressure. The finding of several replacements in the N-terminal portion of the *AgAcp34A-3 *secreted peptide leads us to hypothesize that this region represents the least constrained part of the protein, where the fixation of novel mutations could more easily occur. Finally, other homogenization events among the three genes (e.g. an ectopic recombination, as inferred between *AgAcp34A-1 *and *AgAcp34A-3*) likely contributed to the evolution of these paralogs, complicating the interpretation of their evolutionary patterns: for instance, the degree of sequence variation observed in flanking regions of *AgAcp34A-1 *and *AgAcp34A-2 *is consistent with the hypothesis that these two duplicates originated earlier than *AgAcp34A-3*, whose flanking regions nearly perfectly match those of *AgAcp34A-2*, thus indicating a more recent origin. However, we cannot rule out the hypothesis that *AgAcp34A-3 *could be the actual ancestral gene from which *AgAcp34A-2 *(and, later, *AgAcp34A-1*) would have originated. However, this would have required an extensive homogenization process between *AgAcp34A-2 *and *AgAcp34A-3 *leading to decreased nucleotide diversity in flanking regions, but not in coding regions. Additional data are needed to assess the orthology, synteny and diversity of these genes in other *Anopheles *species and to clarify the sequence of duplication events generating the copies of this gene family.

From a functional perspective, gene duplication is believed to increase the plasticity of transcriptomes [15,40]. In fact, novel copies, being initially free from selective pressure because of their functional redundancy, depending on the relative forces of selection and drift may: i) remain very similar to the original copy and maintain the same function; ii) diverge and acquire a new function (neo-functionalization); or iii) lose their function and become pseudogenes (pseudogenization). Although we do not have direct allele-specific evidence of protein translation, we did not detect mutations in nucleotide sequences of any of the three genes that could impair the ability of some haplotypes to code for a functional protein. Furthermore, we found that all three genes were transcribed in males of at least 3 species (*A. gambiae *s.s., *A. arabiensis *and *A. merus*) (Additional file 1), suggesting that pseudogenization has not occurred. Previous RT-PCR assays had shown that *AgAcp34A-1 *and *AgAcp34A-2 *were exclusively expressed in MAG of *A. gambiae *s.s. [18], although these experiments were not designed to efficiently distinguish among transcripts produced by the three paralogs. Recently, mass spectrometry proteomic analysis demonstrated that *AgAcp34A-2 *(and/or *AgAcp34A-3*, but no data are available to confirm the presence of *AgAcp34A-1*) is among the proteins expressed exclusively in MAG of *A. gambiae *s.s and among the components of the mating plug [20]. Indeed our immuno-fluorescence assays confirmed that an AGAP009370-like protein is specifically expressed in the posterior part of MAG in all *Anopheles *species here examined (Figure 5c, d) and showed that one or both proteins are concentrated at the posterior tip of the *A. gambiae *s.s. mating plug (Figure 5b). As the antibody used in immunofluorescence might recognize both *AgAcp34A-2 *and *AgAcp34A-3*, we cannot state with certainty whether one or both proteins are transferred to females during copulation. Regardless, the specific localization of the AGAP009370-like protein on the portion of the mating plug that is in close proximity to the duct connecting the atrium to the spermatheca suggests two considerations: i) the process of plug formation and transfer is spatially and temporally organized, and ii) the AGAP009370-like protein might play a role in sperm function upon migration to the spermatheca.

In *Drosophila*, the comparisons of gene sequences within and between species have shown that Acps are rapidly evolving and that many paralogs that arose by gene duplication events have diverged rapidly from their ancestral copy under directional selection, leading in some circumstances to the acquisition of lineage-specific duplicates [4-12]. The availability of multiple *Drosophila *genome sequences has allowed evaluation of the evolutionary hypotheses of positive selection on these male-specific reproductive proteins in a robust phylogenetic and functional context. In contrast, the lack of genomic data on *Anopheles *taxa other than *A. gambiae *s.s. and the absence of a reliable phylogenetic background do not allow to infer the evolution of characters along well-defined lineages (e.g. in monophyletic sister-groups) [21,41]. Furthermore, although the *A. gambiae *complex represents an interesting model to study the adaptive evolution of genes potentially involved in reproductive isolation, the pervasive incomplete lineage sorting of alleles among closely related species affects the interpretation of genetic estimates and the application of selection models [21,42]. Similarly to what has been reported for other genes of this complex [21,43-45], we found several haplotypes shared by multiple *A. gambiae *species in all of the three paralogs, due either to introgressive hybridization and/or to retention of ancestral polymorphisms. Despite this, we observed fixed species-specific replacements in at least one gene from each taxon along their geographical distribution, with the exception of *A. gambiae *s.s. (Figures 1 and 4). In *AgAcp34A-2*, one fixed species-specific replacement (i.e. a glutamine (Q) to a glutamic acid (E) change in the QLLQLLQLL sequence motif at the C-terminal) is observed in *A. arabiensis *(Figure 4b). To our knowledge, this represents the first report of an amino acid substitution in a gene positioned in an area of an autosome not affected by chromosomal inversion polymorphisms that clearly distinguishes *A. arabiensis *from *A. gambiae *s.s.. In fact, extensive genetic exchange of autosomal sequences has been frequently reported between these two largely sympatric sibling species [44,46,47]. Since it has been suggested that genes involved in reproductive isolation may be protected against extensive gene flow [44], it is tempting to speculate on a possible role of *AgAcp34A-2 *to the reproductive isolation between *A. gambiae *s.s. and *A. arabiensis*.

In the other two genes, a higher number of fixed substitutions is observed in those species of the complex characterized by a more restricted geographic distribution: *A. merus *shows very high level of differentiation in *AgAcp34A-1, A. melas *in *AgAcp34A-3 *and *A. quadriannulatus *in both genes (Figure 4a, c). As discussed in studies on the molecular evolution of other genes in this species group [21,41,42], genetic drift might have contributed as a major force in the diversification of these geographically more restricted species and would have thus determined the fixation of species-specific substitutions (and, therefore, lineage-sorting).

It is interesting to note that almost all species-specific substitutions are placed in the C- and N-terminal portions of the secreted peptides: since these are the most strongly differentiated regions among the three paralogs, it is likely that these regions might be the least constrained portions of the three proteins. Alternatively, if these substitutions are affecting the protein functions, their fixation might be preferentially explained by positive selection.

The selective forces shaping the evolution of these genes cannot be fully clarified here due to the above cited limits in the application of selection inferences in the *A. gambiae *complex and to the lack of other information to corroborate the possible significance of the observed amino acid replacements (e.g., no structural conserved domains are recognisable for these proteins, neither indications of possible interaction with other molecules/proteins are available). However, our data provide some clues to the evolutionary forces that may have contributed to the diversification of the paralogs. In all three genes most polymorphisms occurred at synonymous rather than at nonsynonymous sites (Table 2). We can thus hypothesize that purifying selection operates to retain the structure and function of these proteins. Moreover, *AgAcp34A-1 *and *AgAcp34A-2 *appear to be more conserved than *AgAcp34A-3 *(Table 2, Figure 3 and 4). This is particularly evident when comparing the levels of genetic polymorphisms in *A. gambiae *s.s. and *A. arabiensis *(Table 2): the average π values computed over all sites are comparable for *AgAcp34A-1 *and *AgAcp34A-2*, ranging from 0.0 to 0.9%, whereas ~2-3 fold higher values are found for *AgAcp34A-3 *(1.7% to 2.9%). One of the main findings of genomic studies is that duplicate genes do not evolve symmetrically [48], i.e. they do not evolve at the same rate, due either to differences in recombination rate or to relaxation of negative selection and/or an increase in positive selection. Since the three genes are located in the same chromosomal area (at least within ~100 kb, the limit of resolution of a tandem duplication by FISH), they should be subject to the same mutation and recombination rates. It is then more plausible to hypothesize a relaxation of negative selection in *AgAcp34A-3*: in fact, the higher π values found at synonymous *versus *non-synonymous sites likely indicate that purifying selection is more relaxed than in the other two paralogs. This implies that, the resulting protein could tolerate a higher number of amino acid changes, but purifying selection still prevents pseudogenization preserving its function. If both AGAP009370-like proteins were expressed in MAG and transferred to females as part of the mating plug, the observed differences in the selective regime between *AgAcp34A-2 *and *AgAcp34A-3 *could be related to different interactions between these factors and proteins expressed in the female lower reproductive tract. In this case a possible specialization into different (but, probably, complementary) functions during the post-mating processes can be hypothesized.

## Conclusions

Proteins in seminal fluids have roles in modulating female behavioral and physiological responses in many insect species [1-3]. A significant number of these proteins evolve rapidly, but the forces driving these patterns are still not well understood. In polyandrous species, such as *Drosophila*, sexual selection and/or conflict due to male-female protein interactions have been hypothesized as the principal mechanisms of rapid divergence of Acps [4-12]. However, other evolutionary mechanisms may play a role in monandrous species and in species with different mating systems [21,49]. Whatever the causes of this unusual pattern of evolution, it has been argued that the analysis of genetic divergence of seminal fluid proteins should be preferentially performed among closely related species to illuminate the selective forces underlying their evolution [1-3]. However, the lack of a strong phylogenetic background for closely-related species prevents mapping adaptively evolving characters through reliable species-trees, thus hindering the interpretation of the observed evolutionary patterns [21,42]. Despite these limits, assessing the polymorphisms and divergence of genes that may alter reproductive behaviours in medically relevant insect species, such as the mosquitoes of the *A. gambiae *complex, is essential to provide information on the genetic variability of molecules that could potentially be exploited to develop strategies aimed at limiting the fertility of malaria vectors in field populations [2,3].

Our results indicate the existence of species-specific products for three Acp-like *A. gambiae *paralogs that may be indicative of unique species-specific regulations and/or functions. In addition, similarly to *Drosophila *Acps, the presence of three duplicated genes likely favoured different rates of evolution for the copies, increasing the probability of recovering rare favorable mutations and allowing the fixation of replacements (e.g. through positive selection) in different lineages, thereby increasing the plasticity of the transcriptome. This pattern is partly similar to what we observed for genes encoding female serine proteases involved in *A. gambiae *s.s. post-mating processes, in which duplication events possibly provided a way to relax selective constraints in some copies, allowing the fixation of novel variants and conferring adaptation of these proteins to different male-derived substrates in diverging lineages [21]. Preliminary genetic data on AGAP009368 (Plugin) provide further indications that different selective forces could act to change the rate of evolution of *A. gambiae *male-expressed genes, putatively as a consequence of different functional phenotypes. In fact, although this gene is placed just ~2 kb apart from the gene cluster here analysed here, it is strongly conserved among all the species of the *A. gambiae *complex (EM, unpublished results), consistent with its essential role in the *A. gambiae *post-mating mechanisms [20].

*Anopheles gambiae *male-expressed proteins that are transferred with the mating plug must interact with proteins of the female lower reproductive tract. Progress in understanding the signaling cascade in the *A. gambiae *reproductive pathways will elucidate the interaction of MAG-expressed proteins with the female counterparts. This knowledge will allow a better evaluation of the relative importance of genes associated with the reproductive isolation and fertility of *A. gambiae *species and will help the interpretation of the observed evolutionary dynamics.

## Authors' contributions

Conceived and designed the experiments: EM, FB, DWR, FC, AdT. Performed the experiments and/or analysed the results of: i) genetic data: EM, FT, IM; ii) *in silico *analyses: EM, DM, IVS; iii) fluorescence *in situ *hybridization: PG, IVS; iv) dissections and RT-PCR: FT, MC, AS, DWR; v) immunofluorescence and confocal analysis: FB, DWR, FC. Wrote the paper: EM, FB, IVS, FC, AdT. All authors contributed to and approved the final manuscript.

## Supplementary Material

Additional file 1 **Detection of paralog-specific transcripts in female and male tissues of *A. gambiae, A. arabiensis *and *A. merus***. Gene-specific nested RT-PCR using cDNA obtained after RNA extraction from the whole body of females (F-WB), male carcasses (M-C) and male accessory glands (M-MAG) as templates. For each tissue, *A. gambiae *products were run in the first lane, *A. arabiensis *products in the second lane and *A. merus *products in the third lane. Genomic DNA (gDNA) was amplified simultaneously to check for the efficiency of nested PCR reactions (e.g., primer annealing efficiency) in all analysed species. Ribosomal protein rpS7 was used to exclude genomic DNA contamination of cDNA templates (expected product size: cDNA = 458 bp, gDNA = 610 bp). Click here for file
