# Supplementary figures and images for: Molecular characterization and evolution of a gene family encoding male-specific reproductive proteins in the African malaria vector Anopheles gambiae
Source: BMC Evol Biol. 2011 Oct 6;11:292. doi: 10.1186/1471-2148-11-292 (PMC3199272; doi:10.1186/1471-2148-11-292)

|  | <i>F</i><br>(W.B.) | <i>M</i><br>(C.) | <i>M</i><br>(MAG) | <i>gDNA</i> |
|--|--------------------|------------------|-------------------|-------------|
|--|--------------------|------------------|-------------------|-------------|

*AgAcp34A-1*

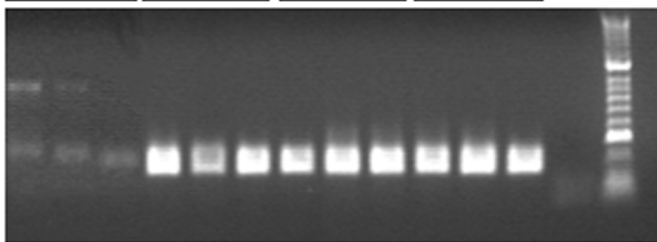

*AgAcp34A-2*

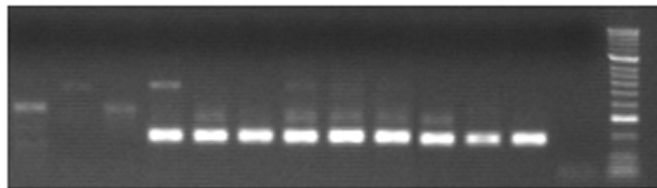

*AgAcp34A-3*

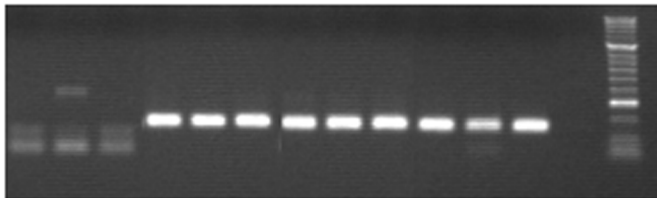

*rpS7*

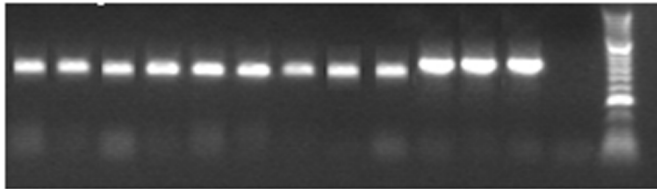

Supplement: Additional file 1 — Detection of paralog-specific transcripts in female and male tissues of A. gambiae, A. arabiensis and A. merus. Gene-specific nested RT-PCR using cDNA obtained after RNA extraction from the whole body of females (F-WB), male carcasses (M-C) and male accessory glands (M-MAG) as templates. For each tissue, A. gambiae products were run in the first lane, A. arabiensis products in the second lane and A. merus products in the third lane. Genomic DNA (gDNA) was amplified simultaneously to check for the efficiency of nested PCR reactions (e.g., primer annealing efficiency) in all analysed species. Ribosomal protein rpS7 was used to exclude genomic DNA contamination of cDNA templates (expected product size: cDNA = 458 bp, gDNA = 610 bp). [file 1471-2148-11-292-S1.PDF]
